# The Effect of Radiotherapy on Ultrasound-Guided Fine Needle Aspiration Biopsy and the Ultrasound Characteristics of Neck Lymph Nodes in Oral Cancer Patients after Primary Treatment

**DOI:** 10.1371/journal.pone.0149346

**Published:** 2016-03-08

**Authors:** Wu-Chia Lo, Po-Wen Cheng, Chi-Te Wang, Pei-Wei Shueng, Chen-Hsi Hsieh, Yih-Leong Chang, Li-Jen Liao

**Affiliations:** 1 Department and Graduate Institute of Pathology, National Taiwan University College of Medicine, Taipei, Taiwan; 2 Department of Otolaryngology, Far Eastern Memorial Hospital, Taipei, Taiwan; 3 Oriental Institute of Technology, Taipei, Taiwan; 4 Division of Radiation Oncology, Department of Radiology, Far Eastern Memorial Hospital, Taipei, Taiwan; Technische Universitaet Muenchen, GERMANY

## Abstract

**Objectives:**

To investigate the effect of radiotherapy (RT) on ultrasound-guided fine needle aspiration (USgFNA) and sonographic characteristics in the assessment of cervical lymph nodes (LNs) in oral squamous cell carcinoma (OSCC) patients after primary treatment.

**Materials and Methods:**

88 treated OSCC patients underwent 111 USgFNAs of the neck LNs after US evaluation. Among them, 48 USgFNAs were performed on 40 patients following RT and 63 USgFNAs on 48 patients without previous RT. The results of USgFNA and the US characteristics were compared between these two groups.

**Results:**

USgFNA had a sensitivity of 88.0%, specificity of 91.4%, positive predictive value (PPV) of 88.0%, negative predictive value (NPV) of 91.4% and accuracy of 90.0% in patients without previous RT, and a sensitivity of 97.1%, specificity of 83.3%, PPV of 94.3%, NPV of 90.9% and accuracy of 93.5% in those with previous neck RT. The ranges of the short-axis and long-axis length were 13.3 ± 8.0 mm (mean ± SD) versus 17.8 ± 9.1 mm, and 18.6 ± 9.0 mm versus 24.4 ± 10.9 mm for recurrent LNs from patients with, versus without, previous RT (both ps < 0.05), respectively. 76.5% (26/34) of the recurrent nodes after RT and 48% (12/25) of the recurrent nodes without previous RT exhibited an irregular margin (p < 0.05). Additionally, irradiated recurrent LNs had a significantly decreased percentage of discernable calcification compared with non-irradiated recurrent nodes (48% versus 20.6%, p < 0.05).

**Conclusions:**

RT had influence on sonographic characteristics but no influence on USgFNA in diagnosing recurrent LNs in treated OSCC patients.

## Introduction

Oral squamous cell carcinoma (OSCC) is endemic in Southeast Asia because of habitual betel nut and chewing tobacco consumption. The primary treatment is surgery with/without adjuvant radiotherapy (RT) or chemoradiation therapy, depending on the stage of the disease [1,2]. The single most important prognostic factor in patients with OSCC is the status of the cervical lymph nodes (LNs) [1–3]. For the subsequent treatment, it is very important to determine whether the LNs are malignant or not. High-resolution ultrasound (US) is a reliable and widely used imaging modality to assess enlarged cervical LNs [4,5]. The reported sonographic features that help to identify metastatic LNs include size, shape, margin, echogenic hilum, vascular pattern and internal echo [4–6]. Additionally, US-guided fine needle aspiration (USgFNA) is widely accepted as an effective method for diagnosing cervical lesions of the head and neck [5,7–10], and it is reportedly superior to palpation-guided FNA in significantly reducing non-diagnostic aspirates [11]. When combined with FNA in the assessment of “head and neck lesions”, this combination had a sensitivity of 90%, specificity of 96%, positive predictive value (PPV) of 96%, negative predictive value (NPV) of 90% and accuracy of 93%, as shown by both a meta-analysis and systemic review of the literature [12]. In precise detail, FNA had a sensitivity of 94.2%, specificity of 96.9%, PPV of 98.9% and NPV of 84.6% in the assessment of cervical “LNs” [13]. Additionally, previous meta-analyses compared the diagnostic accuracy of different imaging modalities in neck node evaluation and showed that USgFNA was more accurate compared to CT, MRI and US [14]. However, neither the effect of previous radiation on the sonographic features nor the diagnostic efficacy of FNA in metastatic nodal disease in OSCC patients has been fully addressed. The potential lowering of the diagnostic efficacy of FNA or altered ultrasonographic features after RT can result in a delay in the diagnosis, which may render a successful salvage operation and led to a worse survival outcome subsequently.

There is only one report in the literature that compared the diagnostic performance of FNA in 67 patients with and without previous irradiation in head and neck cancer patients [15]. It was reported that FNA had a significantly worse sensitivity (40%), NPV (37%) and accuracy (54%) in patients with previous RT [15]. However, the inadequate rate of FNA was quite high in both groups (>26.3%) and it was not mentioned whether the FNA was performed under US guidance. Additionally, the adequate number of FNA procedures in the RT group was relatively small (n = 28) in their series.

Ahuja et al.[16] reported that the largest cervical nodes in nasopharyngeal carcinoma (NPC) patients became significantly smaller size and lesser round in shape after neck irradiation. They also showed that some non-recurrent nodes in NPC patients after irradiation had significantly less present hilum, more echogenic area, less sharp nodal border and less preserved fascial plane compared to normal subjects without previous RT [17]. However, the definition of metastatic LNs in these two series was based on sonographic features alone.

In our institution, OSCC patients who had pathological risk factors received either adjuvant intensity-modulated RT or helical tomography to their neck. For N0 or N positive neck without extracapsular spread, total radiation dose of 45–60 Gy to the neck would be given; whereas for N positive neck with extracapsular spread, total radiation dose of 66–70 Gy would be used. To date, there has been no report comparing the sonographic characteristics of irradiated and non-irradiated malignant LNs in treated OSCC patients. The aim of the present study was to investigate the effect of radiotherapy (RT) on both the role of USgFNA and the sonographic characteristics in diagnosing recurrent lymph nodes in treated OSCC patients.

## Materials and Methods

In July 2007 we set up an online database including all of the morphological US parameters and images recorded with a Marosis PACS system (Marotech Inc., Seoul, South Korea) in all patients underwent head and neck US at a tertiary referral center. Additionally, we routinely scheduled neck US exam when regional recurrence was in doubt or every 3 to 6 months during the first 3-year follow-up in our treated head and neck cancer patients since then. Based on the protocol, all patients underwent a thorough neck exam, including the thyroid and salivary glands, and the neck from level I to level VI, using grey-scale and power Doppler US. Additionally, each patient who was eligible for FNA was performed under the guidance of US. Written informed consent was provided by every patient. The current study protocol was approved by the institutional review board (FEMH IRB 103100-E) of the local ethics committee, at Far Eastern Memorial Hospital. We retrieved all of the online sonographic data from patients with the diagnoses of oral cancer between Jan. 2008 to Dec. 2012. The medical and pathological records of these patients were reviewed. The inclusion criteria were those patients who had completed their initial cancer treatment and had received USgFNA and subsequent neck dissection or follow up for at least 6 months. Final diagnosis was based on cytopatholgic or histopathologic findings. The clinical records of patients with negative cytopatholgic diagnoses were subsequently monitored for a minimum of 6 months to confirm that no malignancy had developed among these LNs. The exclusion criteria included patients with primary oral cancer other than SCC, other malignancies outside the head and neck regions, the presence of a simultaneous second primary cancer, previous RT involving the head and neck regions to treat other diseases and incomplete RT treatment for OSCC. Ultimately, 111 USgFNAs in 88 treated OSCC patients were enrolled and analyzed. These patients were divided into two groups according to whether or not previous neck RT had been performed. In the RT group, 52.5% patients received intensity-modulated RT and the others had helical tomography to their neck.

The sonograms were performed by two sonographers (L.-J.L. and W.-C.L.) using an ATL HDI 5000 with a high-resolution 7.5–12 MHz real-time linear-array transducer (Philips Ultrasound, Bothell, WA, USA). Each of them has had experience with USgFNA in more than 1000 cases. The diameters of the short and long axes, and the ratio of the short to long axes (S/L ratio) of the LNs were measured. The echo structure was categorized as either a predominantly solid component or mixed cyst type. The nodal margin was classified as regular or irregular. Echogenicity with respect to the proximal muscles was assessed and classified as hypoechoic, isoechoic or hyperechoic. Echogenic hilum was distinguished in terms of its presence and absence. Calcification was also distinguished by its presence or absence. The internal echo was inspected for the presence of a homogenous or heterogeneous pattern. The settings for Power Doppler sonography were set for high sensitivity with a low wall filter to allow for the detection of vessels with a low blood flow [18] and the vascular pattern was categorized as being of an avascular or hilar type versus that of a mixed, spotted or peripheral type. Without preceding local anesthesia, USgFNA was performed using the free hand technique by positioning the array probe parallel to the 22-G needle for the purpose of guidance and placement within the neck LNs suspected of malignancy. One aspiration from six passes into the node was performed to obtain sufficient material for cytological evaluation. Cytological staining including Liu and Papanicolaou stains.

Continuous data were expressed as the mean ± SD. The FNA result was defined as “positive” if malignant or atypical cells were present, “negative” if no malignant cells were observed, and “inadequate” if there were insufficient cells, too many RBCs obscured the cells that were present, or cells with obvious crush injury were seen. True positive (TP) was defined as positive on initial cytology and having a final diagnosis of malignancy. True negative (TN) was defined as negative on initial cytology and having a final diagnosis of non-malignancy. False negative (FN) referred to cases with a negative initial cytology, but having a subsequent histopathologic diagnosis of malignancy. False positive (FP) referred to cases with an initially positive cytological diagnosis, but a subsequent histopathologic diagnosis of a lack of malignancy. Univariate comparisons of diagnostic outcomes were calculated with the inadequate cases excluded and performed with χ^2^ tests. The Fisher exact, χ^2^ and nonparametric Mann-Whitney U-tests (M-U test) were used to determine the differences in the clinical parameters (i.e. age, sex, side and the level of occurrence, diameter of the short and long axes, S/L ratio, internal echo, echogenicity, margin, ehchogenic hilus and vascular pattern), as appropriate. The statistical significance was set at *P* < 0.05. All statistical analyses were performed using Stata software, version 12.0 (StataCorp. LP, College Station, TX).

## Results

A total of 111 USgFNAs were obtained from 88 treated OSCC patients (Table 1), including 82 males (93.2%) and 6 females (6.8%). The mean age was 53-years-old, ranging from 23 to 84 years. Among them, the subsites of OSCC were mostly from the tongue and buccal mucosa, accounting for 65.9% (58/88) of the patients. The prevalence of malignant LNs was 53.2% (59/111). Final diagnosis revealed 34 and 25 malignant cervical LNs in patients with and without previous neck RT, respectively.

**Table 1:** A comparison of demographic features in treated OSCC patients between the group without prior neck radiation (RT-) and that with previous neck RT (RT+).

	OSCC RT- (n = 48)	OSCC RT+ (n = 40)	*P*-value
Age (mean ± SD)	52.00 ± 10.46	52.88 ± 10.89	NS
Gender (M/F)	45/3	37/3	NS
Subsite (n, %)			NS
Buccal mucosa	11(22.92)	12(30)	
Tongue	22(45.83)	13(32.5)	
Hard palate	3(6.25)	2(5)	
Lip	1(2.08)	2(5)	
Gingiva	3(6.25)	4(10)	
Mouth floor	3(6.25)	3(7.5)	
Retromolar trigone	5(10.42)	4(10)	

NS: non-significant

A comparison of the diagnostic efficacy of FNA is presented in Table 2. For the results of USgFNAs, the adequate rate for analysis was 95.2% and 95.8% in patients without and with previous neck RT, respectively (p = 0.88). USgFNA had a sensitivity of 88.0%, specificity of 91.4%, PPV of 88.0%, NPV of 91.4% and accuracy of 90.0% in patients without previous RT, and a sensitivity of 97.1%, specificity of 83.3%, PPV of 94.3%, NPV of 90.9% and accuracy of 93.5% in those with previous neck RT. No significant difference was detected between the two groups in any of the diagnostic parameters. In the “positive” FNAs, there were 7 and 8 suspicious reports (atypical cells) in patients with and without previous neck RT, respectively. Final diagnoses showed that in 7/7 and 5/8 FNAs had true malignancy in the groups with and without preceding neck irradiation, respectively. The 3 false positive cases in the latter group were confirmed by negative neck dissection. In the rest of the “positive” FNAs, all proved to be malignant in patients without RT, whereas 2 cases, who harbored no nodal metastasis from the neck dissection, had false positives in the neck RT group.

**Table 2:** A comparison of the diagnostic efficacy of USgFNA in treated OSCC patients without previous neck RT (RT-) and those with prior neck irradiation (RT+).

	OSCC RT-	OSCC RT+	*P*-value^#^
Adequate rate	95.2% (60/63)	95.8% (46/48)	0.88
Sensitivity (95% CI)	88.0% (75.3%~100.7%)	97.1% (91.4%~102.7%)	0.40
Specificity (95% CI)	91.4% (82.2%~100.7%)	83.3% (62.2%~104.4%)	0.81
PPV (95% CI)	88.0% (75.3%~100.7%)	94.3% (86.6%~102.0%)	0.69
NPV (95% CI)	91.4% (82.2%~100.7%)	90.9% (73.9%~107.9%)	0.96
Accuracy (95% CI)	90.0% (82.4%~97.6%)	93.5% (86.3%~100.6%)	0.78

^#^: χ^2^ test

We then examined which of the demographic or clinical variables were associated with malignant LNs in each group. Malignant LNs were significantly associated with increased short- and long axes diameters, heterogeneous internal echo, irregular margin and absent echogenic hilum, but were not significantly related to patient age, sex, or tumor laterality or level in the group with (Table 3) or without previous neck RT. The sonographic features of the enlarged S/L ratio as well as calcification were significantly associated with malignancy in the LNs without previous RT; however, these two features displayed no significant difference in LNs following neck irradiation.

**Table 3:** A comparison of demographic features between benignity and malignancy in treated OSCC patients with previous neck RT (RT+).

Factors	RT+ benignity (n = 14)	RT+ malignancy (n = 34)	*P*-value
Age (Y)*	51.6±8.9	54.3±11.5	0.213^§^
Gender (F/M)	0/14	3/31	0.251^#^
Side (L/R/B)	9/4/1	16/15/3	0.548^#^
Site			0.864^£^
Level 1 (30.51%)	3	7	
Level 2 (33.90%)	5	10	
Level 3 (15.25%)	2	8	
Level 4 (3.39%)	1	2	
Level 5 (10.17%)	3	4	
Level 6 (6.78%)	0	3	
Site (Level 1–3/4–6)	10/4	25/9	1.000^£^
Short-axis diameter (cm)*	0.68±0.3	1.33±0.8	<0.001^§^
Long-axis diameter (cm)*	1.18±0.5	1.86±0.9	0.013^§^
S/L ratio*	0.62±0.2	0.76±0.3	0.188^§^
Internal echo (homo/hetero-geneous)	10/4	8/26	0.003^£^
Echogenic level (hypo/iso/hyper-echogeneicity)	13/0/1	32/0/2	1.000^£^
Margin (regular/irregular)	12/2	8/26	0.000^£^
Echogenic hilum (present/absent)	6/8	4/30	0.045^£^
Calcification (present/absent)	0/14	7/27	0.090^£^
Architecture (solid/cystic)	13/1	25/9	0.242^£^
Vascular pattern (avascular or hilar/others)	12/2	24/8	0.701^£^

*:Data are presented as mean ± SD

^§^: Mann-Whitney U-test

^#^: χ^2^ test

^£^: Fisher-exact test

**Fig 1:**
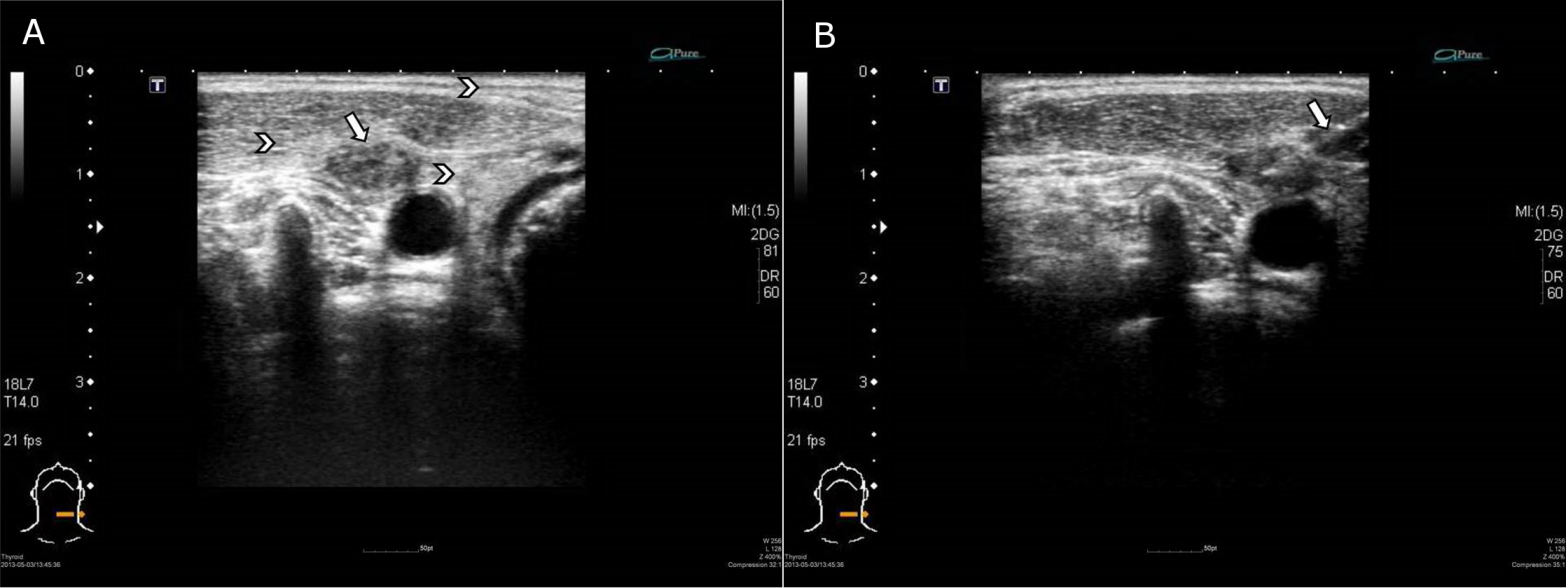
A 54-year old man who had a recurrent neck LN at left level IV after neck irradiation. (A)The left side ultrasound image showed that this metastatic LN was small, with an irregular margin and had no detectable calcification. Increased echogenicity of subcutaneous layer, overlying muscle and perinodal area was also noted. (arrow indicated irregular boundary and arrowheads showed increased echogenicity) (B)The right side image shows the USgFNA of the node. (arrow designated the needle)

**Fig 2:**
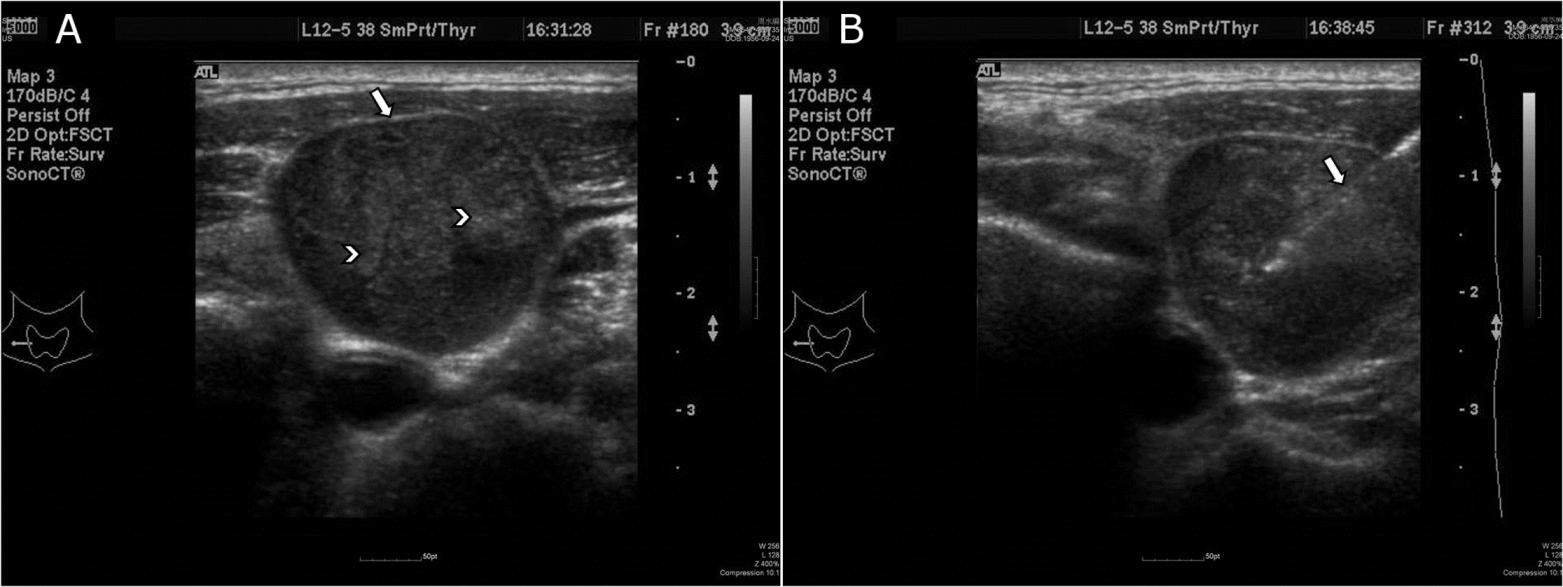
This 54-year-old man had a recurrent LN at right level III without previous neck irradiation. (A)The left side ultrasound image showed that this recurrent LN was large with a regular margin and had detectable calcification. (arrow indicated regular boundary and arrowheads indicated microcalcification) (B)The right side image shows the USgFNA of the node. (arrow designated the needle)

We further compared various sonographic parameters in malignant LNs between treated OSCC patients with and without preceding neck RT (Table 4). Malignant nodes were distributed mainly in levels I and II of the neck in patients without RT. However, in patients receiving adjuvant RT, the recurrent nodes were most located at levels I to III of the neck. Intriguingly, there were 3 cases of recurrent metastatic LNs in level VI after RT, a result which was not found in treated OSCC patients without previous RT. There was no difference in the final pathologic nodal stage between the two groups. However, the range of short-axis diameter was 13.3 ± 8.0 mm for malignant nodes in patients with previous irradiation, significantly smaller than that (17.8 ± 9.1 mm) in patients without RT (p < 0.05). The length of long-axis diameter was 18.6 ± 9.0 mm versus 24.4 ± 10.9 mm for malignant LNs in patients with and without previous RT, respectively (p < 0.05). There was no difference in the S/L ratio of the malignant nodes between the two groups (p = 0.33). Metastatic LNs in both groups exhibited a round shape rather than a fusiform contour. Irradiated malignant LNs had a significantly decreased percentage of distinguishable calcification compared with non-irradiated malignant nodes (48% versus 20.6%, p < 0.05). Additionally, irradiated metastatic nodes had significantly more irregular margin features than non-irradiated ones (76.5% versus 48%, p < 0.05). In summary, the recurrent cervical nodes in treated OSCC patients with previous neck RT (Fig 1A and 1B) were of smaller size, had reduced discernible calcification and more signs of an irregular margin on US than those without previous RT (Fig 2A and 2B).

**Table 4:** A comparison of demographic features of malignancy in treated OSCC patients without previous neck RT (RT-) and those with prior neck irradiation (RT+).

Factors	RT- malignancy (n = 25)	RT+ malignancy (n = 34)	*P*-value
Age (Y)*	52.3±10.3	54.3±11.5	0.242^§^
Gender (F/M)	1/24	3/31	0.466^#^
Side (L/R/B)	13/10/2	16/15/3	0.932^#^
Final pN stage			0.659^£^
pN1	9	12	
pN2	15	22	
pN3	1	0	
ECS (present/absent)	18/7	30/4	0.176^£^
Site			0.112^£^
Level 1 (30.51%)	11(44%)	7(20.59%)	
Level 2 (33.90%)	11(44%)	10(29.41%)	
Level 3 (15.25%)	1(4%)	8(23.53%)	
Level 4 (3.39%)	2(8%)	2(5.89)	
Level 5 (10.17%)	0	4(11.76%)	
Level 6 (6.78%)	0	3(8.82%)	
Site (Level 1–3/4–6)	23/2	25/9	0.207^£^
Short-axis diameter (cm)*	1.78±0.9	1.33±0.8	0.019^§^
Long-axis diameter (cm)*	2.44±1.1	1.86±0.9	0.034^§^
S/L ratio*	0.73±0.2	0.76±0.3	0.613^§^
Internal echo (homo/hetero-geneous)	6/19	8/26	0.967^#^
Echogenic level (hypo/iso/hyper-echogeneicity)	24/0/1	32/0/2	1.000^£^
Margin (regular/irregular)	13/12	8/26	0.047^#^
Echogenic hilum (present/absent)	0/25	4/30	0.130^£^
Calcification (present/absent)	12/13	7/27	0.026^#^
Architecture (solid/cystic)	20/5	25/9	0.564^#^
Vascular pattern (avascular or hilar/others)	17/8	24/8	0.559^#^

*:Data are presented as mean ± SD

^§^: Mann-Whitney U-test

^#^: χ^2^ test

^£^:Fisher-exact test

## Discussion

A history of RT of the neck is commonly encountered during the management of OSCC. The induration that occurs due to fibrosis of the muscles and the soft tissue of the neck after RT may mask an underlying lymphadenopathy that is easily missed by palpation alone. This scenario can impede an early diagnosis of residual or recurrent neck node metastasis. Although magnetic resonance imaging (MRI), computed tomography (CT), positron emission tomography (PET) and US are commonly used to evaluate cervical LNs, physicians are still in need of a determination of the LN content when malignancy is doubted. FNA under US guidance is particularly useful in assessment of the neck for this group of patients and is often performed as an integral technique in the pre-operative investigation. It is fast, safe, inexpensive, relatively noninvasive with few complications, and a well-established technique for the diagnosis of malignant cervical LNs, especially carcinoma [4,5,19–21]. In a systematic review and meta-analysis, the pooled sensitivity, specificity, PPV and NPV for cervical LNs were 94.2%, 96.9%, 98.9% and 84.6%, respectively [13]. However, few studies have been performed to determine the diagnostic efficacy in cervical LNs following RT, despite its common utilization.

In the literature, there is only single paper that compared the diagnostic efficacy of needle aspiration in 67 head and neck cancer patients with and without previous RT [15]. Chan et al.[15] reported a meaningfully worse sensitivity (40%), NPV (37%) and accuracy (54%) of FNAs in the RT group. Nonetheless, it was not stated whether the FNA was accomplished under US guidance or not. Also, the FNAs in the RT group were relatively small and the inadequate rate was comparatively high in the study. Nishimura et al.[9] reported that results with irradiated neck LNs in patients with head and neck SCC using FNA were quite good, with a sensitivity of 71.4% and specificity of 95.6%. However, the authors did not compare with patients without neck RT.

To the best of our knowledge, the present study is the first report to investigate the diagnostic efficacy of FNA solely in treated OSCC patients with a large number of cases. The sensitivity, specificity, PPV and NPV of FNA in treated OSCC patients without RT were all approximately 90%, which corresponds to the findings of meta-analysis studies [12,13]. Surprisingly, the diagnostic efficacy of FNA in treated OSCC patients with prior irradiation has a comparable adequacy rate, sensitivity, specificity, PPV, NPV and accuracy. It is well documented that cancer cells may be arranged as scattered infiltrative nests intermixed with fibrous stroma, which might hamper the chance of correct sampling and increase the possibility of FN results [22]. For irradiated NPC patients, it is also reported that FP results of palpation-guided FNA may lead to negative neck dissection outcomes [23]. However, we found similar high adequate rates, and low FN and FP rates of USgFNA in both groups. We believe the sampling errors would be diminished considerably under the guidance of US performed by experienced operators. In the present study, the diagnostic value of FNA in the RT group is similar to Nishimura et al.[9], but superior to Chan et al.[15]. This result is likely due to differences in the inclusion criteria, experience of the cytopathologists, FNA setting and operator technique in the different studies. It has been shown that the combination of a variety of diagnostic tests provides a significant improvement in the accuracy of detecting metastatic disease [24]. For treated OSCC patients, we demonstrated that USgFNA exhibits a comparable and excellent diagnostic efficacy for irradiated and non-irradiated neck nodes. As a result, we recommend using USgFNA for a determination of nodal malignancy if one imaging modality indicates regional recurrence.

As far as we are aware, there has been no previous report comparing the sonographic characteristics of irradiated and non-irradiated malignant LNs in treated OSCC patients. It has been shown that after RT, some lymphoid tissues are missing and part of LN was replaced by fibrous tissue [22]. The diagnostic criteria in the detection of pathologic LNs such as nodal necrosis or a short axis diameter on CT scan in irradiated neck are reportedly sometimes unreliable [25]. In NPC patients, Ahuja et al.[16] reported that the largest cervical nodes became significantly smaller and less round in shape after neck irradiation under US. As shown in Table 4, our data shows that the diameter of both the S- and L-axes was smaller in irradiated than non-irradiated recurrent LNs, although both groups had a similar status of nodal pathology and shape. This finding is consistent with the hypothesis that, with a dampened effect the LN infiltrated by cancer cells is extended to a lesser degree than when it is not irradiated [15]. Additionally, we showed that recurrent cervical nodes in treated OSCC patients with previous RT had significantly less calcification. This is probably because the nodes and surrounding soft tissue or the overlying muscle become more echogenic after neck irradiation [17], and that the size of recurrent LNs was smaller in the irradiated group, both of which may influence the evaluation of calcification. Furthermore, an increasingly irregular margin was detected in the irradiated than non-irradiated recurrent nodes. This result is consistent with the finding that the nodal border became less sharp following RT, as reported by Ahuja et al [17]. After the irradiation, not only may recurrent cancer cells become rearranged as scattered infiltrative nests within LNs, but also the nodal capsule is sometimes replaced by fibrous tissue, either or both of which may result in an irregular margin.

The roles of US and USgFNA are not only for detection and biopsy of LN prior and after radiation treatment. High-intensity focused US, which means high-frequency US waves produced by transducer are directionally focused on the target region within the body, is a minimally invasive energy ablation technique. The thermal and mechanical effects can cause coagulative necrosis of the tumor. It is reported to facilitate treatment of prostate cancer, gastric cancer, pancreatic cancer, and hepatocellular carcinoma [26–29]. Additionally, high-intensity focused US could not only cause coagulation necrosis but also enhance apoptosis and growth inhibition in SCC xenograft models [30,31]. Furthermore, nanoparticles containing chemotherapy pharmaceuticals or radiopharmaceutical agents offer novel routes to the biofunctionalization for targeting malignant cells [32,33]. Currently, if salvage neck dissection is not feasible when a recurrent LN is diagnosed, re-irradiation and/or chemotherapy are often the only treatment choice for the patient. Hopefully applicability of high-intensity focused US and/or the USgFNA method in combination with nanoparticles is promising for OSCC therapy in the near future.

Certain limitations of this study should be noted. First, in both groups, some of the patients had undergone elective or comprehensive neck dissection. Consequently, the neck level of the recurrent LNs may have been influenced by the preceding neck operation. Second, selection bias may have played a role, because only those patients who had received US and USgFNA were included in the present study. Finally, not all of our final diagnoses were based on the results of histopathologic findings. After an at least 6-month follow-up, the patient who had benign cytopathologic diagnosis was deemed negative. Inevitably, there was a small chance that the node was malignant when a slow-growing neck metastasis was encountered.

In conclusion, whether patients underwent RT or not before needle aspiration, USgFNA displayed excellent diagnostic efficacy in OSCC patients after primary treatment. The ultrasonographic features that help to identify recurrent nodal disease following RT are an increased short- and long-axis diameter, heterogeneous internal echo, irregular margin and lack of echogenic hilum. Recurrent cervical lymphadenopathies in treated OSCC patients following neck RT had a smaller size, less of a percentage of distinguishable calcification and increased signs of an irregular margin than those without previous RT.
